# Comparison of the pressure-relieving properties of various types of forefoot pads in older people with forefoot pain

**DOI:** 10.1186/1757-1146-7-18

**Published:** 2014-03-05

**Authors:** Pei Y Lee, Karl B Landorf, Daniel R Bonanno, Hylton B Menz

**Affiliations:** 1 Department of Podiatry, Faculty of Health Sciences, La Trobe University, Bundoora, Victoria 3086, Australia; 2 Lower Extremity and Gait Studies Research Program, Faculty of Health Sciences, La Trobe University, Bundoora, Victoria 3086, Australia

**Keywords:** Aged, Pain, Forefoot, Orthotic devices, Gait, Kinetics

## Abstract

**Background:**

Plantar forefoot pain is commonly experienced by older people and it is often treated with forefoot pads to offload the painful area. However, studies have found inconsistent effects for different forefoot pads on plantar pressure reduction, and optimum forefoot pad placement is still not clear. The aim of this study was to compare the effects of different forefoot pads on plantar pressure under the forefoot in older people with forefoot pain.

**Methods:**

Thirty-seven adults (31 females, 6 males) with a mean age of 73.5 (SD 4.8) participated. Forefoot plantar pressure data were recorded using the pedar®-X in-shoe system while participants walked along an 8 m walkway. Five conditions were tested in a standardised shoe: (i) no padding (the control), (ii) a metatarsal dome positioned 10 mm proximal to the metatarsal heads, (iii) a metatarsal dome positioned 5 mm distal to the metatarsal heads, (iv) a metatarsal bar, and (v) a plantar cover.

**Results:**

Compared to the shoe-only control condition, each of the forefoot pads significantly reduced forefoot peak pressure and maximum force. The metatarsal dome positioned 5 mm distal to the metatarsal heads and the plantar cover were most effective for reducing peak pressure (17%, *p* < 0.001 and 19%, *p* < 0.001, respectively).

**Conclusions:**

These findings indicate that forefoot pads are effective for reducing forefoot pressures in older people with forefoot pain, and that the position of the pad relative to the metatarsal heads may be more important than the shape of the pad.

## Introduction

Foot pain in older people is associated with decreased mobility [1], self-reported disability [2], impaired balance [1], an increased risk of falling [3] and decreased health-related quality of life [2,4]. The most commonly reported location of foot pain in older people is the forefoot [2,4,5], with prevalence estimates ranging from 20 to 25% [2,5]. In older people, forefoot pain is commonly associated with toe deformity and plantar hyperkeratosis [1,5,6]. These conditions have been associated with increased plantar pressures [7-9], and higher plantar pressures have been associated with foot pain [3,10-13].

Plantar forefoot pain is often treated with forefoot pads [13-16], which are thought to be effective by reducing plantar pressure under the metatarsal heads [13,14,17]. Such reductions are commonly thought to occur by redistributing plantar forces across a larger area of the foot [13,18-20]. However, a variety of forefoot pad designs exist [15,16] and studies have found inconsistent results when evaluating the effectiveness of different forefoot pads for reducing plantar pressures [13,17]. Furthermore, the best placement of the forefoot pad is still not clear, as a range of different positions have been recommended, and improper placement may actually increase plantar pressures under the forefoot [21-23].

With the above in mind, further research is required to determine the most appropriate forefoot pad design and placement to alleviate forefoot pain. Therefore, this study aimed to compare the effects of different forefoot pads on plantar pressure under the forefoot in older people with forefoot pain.

## Methods

### Participants

An *a priori* sample size calculation using an appropriate formula [24] determined that a sample size of 25 would provide an 80% probability of detecting a clinically worthwhile difference between interventions of 60 kPa (SD = 75 kPa, α = 0.05) in peak plantar pressure. For this calculation, plantar pressure data were taken from a similar study that measured plantar pressures in older people [25]. A total of 37 participants were finally recruited for this study.

All participants were recruited from a study population involved in a previous randomised trial [26,27]. Potential participants were posted a letter inviting them to participate, which they were sent at least 9 months after they had completed the randomised trial. Participants were included if they were: (i) community dwelling, (ii) aged 65 years or over, and (iii) had forefoot pain or a previous history of forefoot pain. Participants were excluded from the study if they: (i) were unable to walk household distances (10 metres) without the use of a walking aid, (ii) had any self-reported neurological condition that may have affected their lower limb muscle strength, (iii) had any lower limb surgery in the previous three months, and (iv) were unable to speak basic English. Ethics approval was granted from the La Trobe University Faculty of Health Sciences Human Ethics Committee (Reference FHEC11/143). Written informed consent was obtained from all participants prior to the study.

### Interventions

A standard, lace-up shoe of sole hardness Shore A40 was used for all testing. The shoe was an extra-depth shoe (Gadean®, Perth, Australia), which is commonly prescribed to older people with foot deformity and pain. When used as the control condition, no padding was applied to the inside of the shoe (i.e. the shoe only). Four forefoot plantar pads were tested against the control condition. To allow for appropriate positioning, the pads were adhered to a thin, flexible cardboard template that was the same shape and size as the insole of the shoe. The control condition was also tested with this template, so the only difference between conditions was the type of forefoot pad.

**Figure 1:**
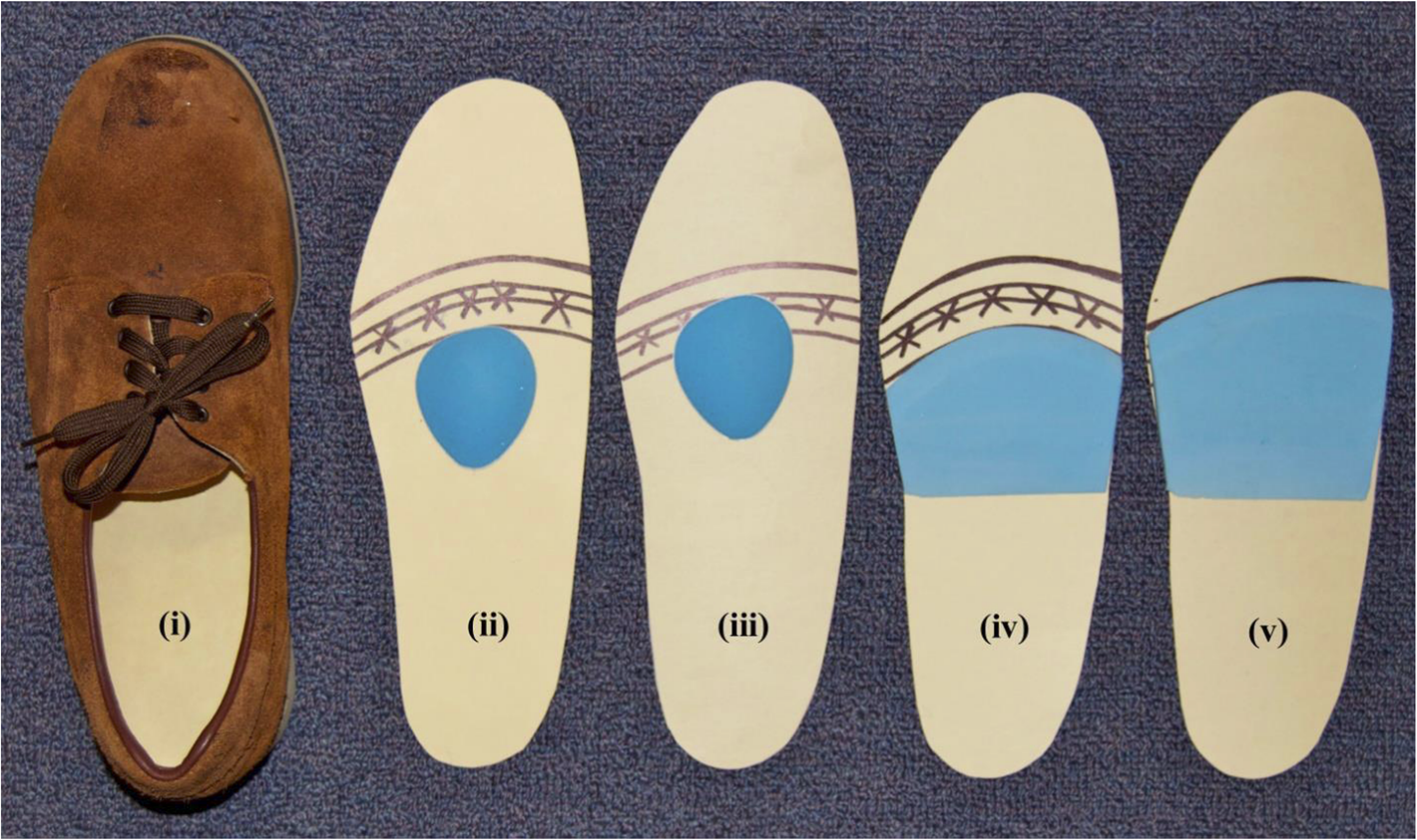
**The interventions used in the study positioned on the cardboard template with metatarsal head position marked by an ‘x’.** Left to right: **(i)** control–shoe only, no padding, **(ii)** metatarsal dome positioned 10 mm proximal to the metatarsal heads, **(iii)** metatarsal dome positioned 5 mm distal to the metatarsal heads, **(iv)** metatarsal bar, **(v)** plantar cover.

The five conditions analysed were (Figure 1):

(i) Control–shoe only, no padding;

(ii) Metatarsal dome placed 10 mm proximal to the metatarsal heads;

(iii) Metatarsal dome placed 5 mm distal to the metatarsal heads;

(iv) Metatarsal bar;

(v) Plantar cover.

To ensure similar thickness, all forefoot pads were made from 6 mm PPT^®^ (Langer Biomechanics, New York, USA). The metatarsal bar and the plantar cover were fabricated by one of the authors (PYL) using a sheet of 6 mm PPT^®^. The metatarsal dome tested was the prefabricated medium sized teardrop-shaped “Metatarsal Pad” manufactured by Langer Biomechanics, which measures 6 mm at its thickest point. The forefoot pads were sized to fit predetermined anatomical landmarks of the participants’ feet to reflect clinical practice (Table 1). All of the plantar cover and metatarsal bar pads were fabricated for a range of shoe sizes (size 4-11) prior to the data collection session. Further details of the fabrication and fitting process for the pads are included in Additional file 1. If a forefoot pad did not fit a participant’s foot during data collection, it was modified accordingly to match the anatomical landmarks of that participant’s foot.

**Table 1:** Borders and dimensions of the forefoot pads

**Forefoot pad**	**Proximal border**	**Distal border**	**Medial border**	**Lateral border**	**Other features**
Metatarsal dome 10 mm proximal^†^	10 mm distal to the styloid process of the 5^th^ metatarsal	10 mm proximal to the metatarsal heads	Medial margin of the 2^nd^ metatarsal	Lateral margin of the 4^th^ metatarsal	Teardrop-shaped
Metatarsal dome 5 mm distal^†^	25 mm distal to the styloid process of the 5^th^ metatarsal	5 mm distal to the metatarsal heads	Medial margin of the 2^nd^ metatarsal	Lateral margin of the 4^th^ metatarsal	Teardrop-shaped
Metatarsal bar	10 mm distal to the styloid process of the 5^th^ metatarsal	10 mm proximal to the metatarsal heads	Medial margin of the 1^st^ metatarsal	Lateral margin of the 5^th^ metatarsal	
Plantar cover	10 mm distal to the styloid process of the 5^th^ metatarsal	15 mm distal to the metatarsal heads	Medial margin of the 1^st^ metatarsal	Lateral margin of the 5^th^ metatarsal	Full thickness (6 mm) under the metatarsal heads

^†^The same metatarsal dome was used in both conditions.

The forefoot pads were adhered using double-sided adhesive tape to the cardboard template to prevent the forefoot pad from moving during testing. To position the pads, the metatarsal heads of the participants were palpated and the centre of the most distal aspect of each metatarsal head was marked on the plantar surface of the foot using a pen. Following this, the template was positioned in the shoe and the participant’s foot was then placed in the shoe on top of the template without socks or stockings. After the shoe was fastened, the participants were instructed to stand to transfer the ink markings onto the template. The metatarsal parabola was then marked on the template and the forefoot pads were positioned accordingly (Figure 1). To ensure consistency, determination of the metatarsal heads and the placement of the forefoot pads for all participants were done by the same investigator (PYL).

### Randomisation and blinding

To minimise ordering effects associated with the administration of the interventions, the order for testing each condition was randomised. Participants were blind to the order of interventions.

### Pressure analysis equipment

Plantar pressures beneath the foot were measured using the pedar®-X in-shoe plantar pressure system (Novel GmbH, Munich, Germany), which has been demonstrated to be a valid and reliable in-shoe pressure measurement system [28-30]. The pedar^®^-X comprises of 99 capacitive sensors arranged in a grid and embedded within a thin flexible insole approximately 2 mm thick. The sampling frequency was 50 Hz. The insoles were calibrated using the trublu^®^ calibration device (Novel GmbH, Munich, Germany) prior to data collection.

### Protocol

The pedar®-X insoles were placed within each shoe between the foot and the forefoot pad to be tested for each forefoot pad condition. The pressure insoles were zeroed as described by the manufacturer’s guidelines (Novel GmbH, Munich, Germany) prior to the first walking trial of each condition. After a familiarisation period of approximately two minutes, the participants completed four walking trials for each condition. Participants were timed as they walked at a comfortable self-selected speed along an 8 metre walkway. To ensure consistency of walking speed, any trial was eliminated and repeated if the time differed by more than 5% of the original trial time. The middle four steps of each trial from the most painful foot were used in the data analysis.

### Outcome measures

The primary outcome measure was peak pressure under the forefoot. Secondary outcome measures were maximum force and contact area under the forefoot.

### Data analysis

**Figure 2:**
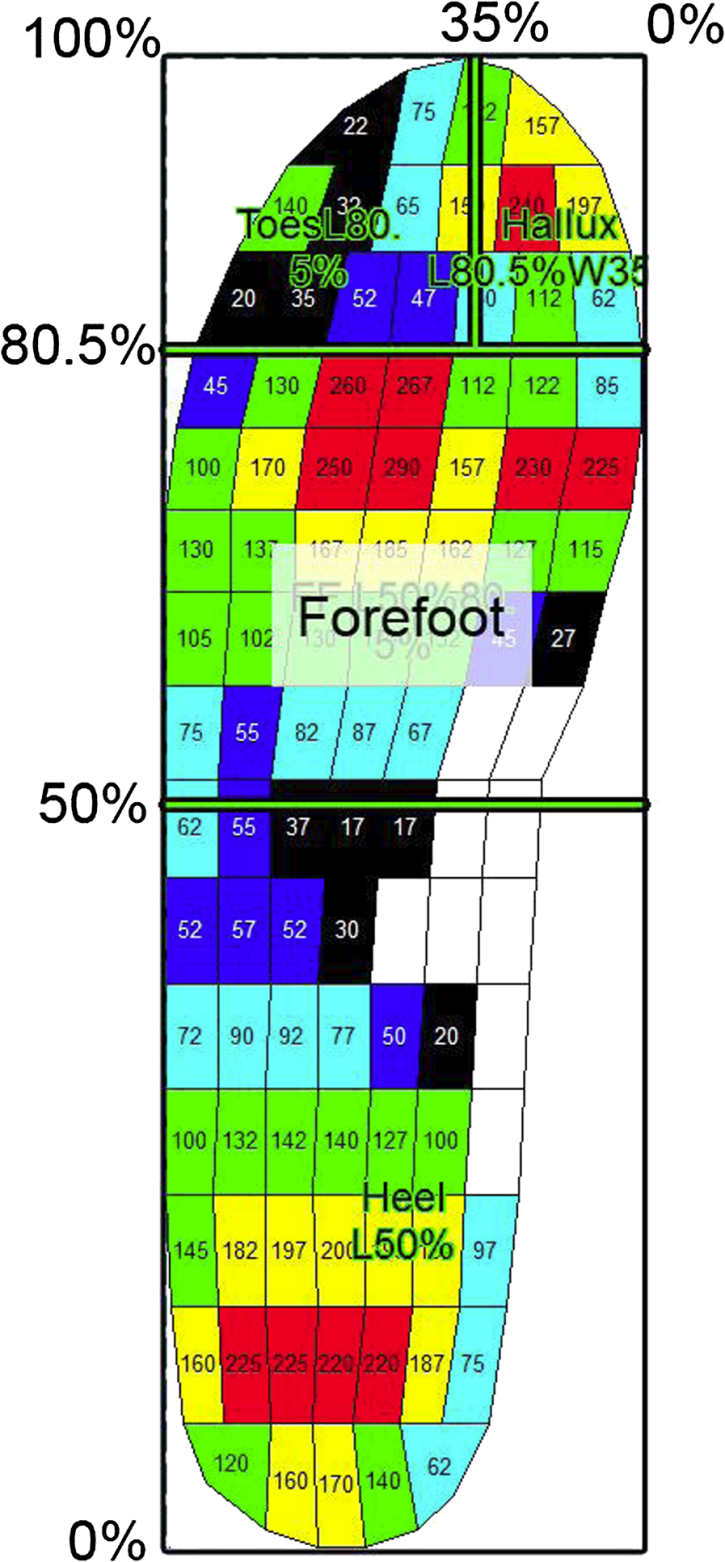
Masks used for the data analysis.

The 16 steps (4 steps x 4 trials) for each condition were averaged and analysed using the Novel-win program (version 20.3.30). Percentage masks were applied to each averaged step, with the forefoot mask being either 31% (small and large pedar^®^-X insoles) or 30.5% (medium pedar®-X insole) of the total foot length (Figure 2). This ensured that the distal aspect of the forefoot mask was at the junction between two rows of sensors in the forefoot and that all of the sensors of the distal aspect of the metatarsal region of the forefoot were fully included in the mask. The 0.5% difference in the percentage mask between the pedar^®^-X insoles was not considered to affect the validity of the pooled data as peak pressure values were based on the size of each individual sensor unit [31] and the masking ensured a complete row of sensor units in the distal forefoot. Moreover, because the masking remained constant for each participant and this study was interested in within-participant comparisons, this adjustment was considered appropriate.

All statistical analyses were performed using SPSS Version 19.0 (SPSS Inc., Chicago, IL, USA). The data were explored for normality prior to inferential analysis. A one-way analysis of variance (ANOVA) with Bonferroni-adjusted post-hoc tests was used to compare means between each of the interventions, with differences considered significant when *p* < 0.05. Where the data violated the assumption of sphericity in ANOVA (if *p* < 0.05 for Mauchley’s Test of Sphericity), the Greenhouse–Geisser correction was used to obtain the degrees of freedom and *p* values for the *F*-statistic.

## Results

Of the 37 participants recruited into the study, 31 were female (84%) and 6 were male (16%). The mean age of participants was 74 with a range of 67 to 87 years. Participant characteristics are shown in Table 2.

**Table 2:** Participant characteristics (N = 37)

**Characteristic**	**Mean (SD), unless otherwise stated**	**Range**
Age in years	73.5 (4.8)	67-87
Females, n (%)	31 (84)	N/A
Height in metres	1.62 (0.06)	1.50-1.78
Weight in kg	77.9 (13.5)	53.6-107.9
Body mass index in kg/m^2^	29.2 (4.2)	22.8-40.3
Current forefoot pain, n (%)	25 (68)	N/A

Overall contact times did not differ across the conditions (Table 3). Therefore, it can be assumed that the participants walked at a consistent speed during the trials and that the observed plantar pressure differences were attributed to the interventions. A number of significant differences in forefoot plantar pressure variables were found between the five conditions (Table 4).

**Table 3:** Total contact time (ms) for each condition (N = 37)

**Condition (forefoot pad)**	**Mean (SD)**	**% change**	***P*** **value**
Shoe only–control	707.0 (75.4)	N/A	N/A
Metatarsal dome 10 mm proximal	709.5 (70.0)	+0.4%	1.000
Metatarsal dome positioned 5 mm distal	719.6 (80.9)	+1.8%	0.656
Metatarsal bar	704.7 (73.6)	-0.3%	1.000
Plantar cover	710.7 (75.8)	+0.5%	1.000

Note: % change is relative to the shoe only–control condition.

**Table 4:** Plantar pressure data for the forefoot (N = 37)

**Condition (forefoot pad)**	**Peak pressure (kPa)**			**Maximum force (%BW)**			**Contact area (cm**^**2**^**)**		
	**Mean (SD)**	**% change**	***P*** **value**	**Mean (SD)**	**% change**	***P*** **value**	**Mean (SD)**	**% change**	***P*** **value**
Control–shoe only, no padding	399.0 (117.6)	N/A	N/A	86.3 (11.3)	N/A	N/A	47.5 (4.9)	N/A	N/A
Metatarsal dome 10 mm proximal	364.7 (98.8)	-9%*	0.004	82.8 (10.4)	-4%*	0.024	50.4 (3.6)	+6%*	0.002
Metatarsal dome positioned 5 mm distal	331.9 (100.3)	-17%*	<0.001	81.7 (11.2)	-5%*	0.006	49.2 (4.3)	+4%	0.176
Metatarsal bar	358.2 (110.4)	-10%*	<0.001	81.3 (11.1)	-6%*	0.001	53.7 (3.6)	+13%*	<0.001
Plantar cover	322.0 (80.6)	-19%*	<0.001	80.7 (7.9)	-6%*	<0.001	51.8 (5.0)	+9%*	<0.001
Metatarsal dome 10 mm proximal *vs* metatarsal dome 5 mm distal	–	-9%	<0.001	–	-1%	1.000	–	-2%	0.055
Metatarsal dome 10 mm proximal *vs* metatarsal bar	–	-2%	1.000	–	-2%	0.114	–	+7%	<0.001
Metatarsal dome 10 mm proximal *vs* plantar cover	–	+12%	0.007	–	-3%	0.111	–	+3%	0.070
Metatarsal dome 5 mm distal *vs* metatarsal bar	–	+8%	0.007	–	0%	1.000	–	+9%	<0.001
Metatarsal dome 5 mm distal *vs* plantar cover	–	-3%	1.000	–	-1%	1.000	–	+5%	<0.001
Metatarsal bar *vs* plantar cover	–	-10%	0.027	–	-1%	1.000	–	-4%	0.003

*Mean difference significant at the 0.05 level (Bonferroni adjusted) compared to the shoe only–control condition.

### Peak pressure

Compared to the control condition, each of the forefoot pads significantly reduced the primary outcome measure of forefoot peak pressure (F_2.6, 93.3_ = 18.6, *p* < 0.01). The metatarsal dome positioned 5 mm distal to the metatarsal heads and the plantar cover resulted in the largest decrease in forefoot peak pressure compared to the control condition: 17% (*p* < 0.001) and 19% (*p* < 0.001) respectively. Both the metatarsal dome positioned 10 mm proximal to the metatarsal heads and the metatarsal bar reduced forefoot peak pressure to a lesser extent compared to the control condition: 9% (*p* < 0.001) and 10% (*p* = 0.004) respectively. Post-hoc pairwise comparisons revealed significant differences between the two groups of conditions that reduced peak pressure to similar extents (the metatarsal dome positioned 5 mm distal to the metatarsal heads and the plantar cover *versus* the metatarsal dome positioned 10 mm proximal to the metatarsal heads and the metatarsal bar) (Table 4).

### Maximum force

Compared to the control condition, each of the forefoot pads significantly reduced maximum force (F_2.8, 99.5_ = 11.5, *p* < 0.05) to a similar extent in the forefoot. Reductions in maximum force by the forefoot pads were similar and ranged from 4% to 6%. Post-hoc pairwise comparisons did not reveal any significant differences between the forefoot pads (Table 4).

### Contact area

Compared to the control condition, each of the forefoot pads except for the metatarsal dome positioned 5 mm distal to the metatarsal heads significantly increased contact area (F_2.8, 100.0_ = 36.5, *p* < 0.01). The metatarsal bar registered the highest increase in contact area (13%, *p* < 0.001) when compared to the control condition, followed by the plantar cover (9%, *p* < 0.001) and then the metatarsal dome positioned 10 mm proximal to the metatarsal heads (6%, *p* = 0.002). Between the two conditions that increased contact area the most, post-hoc pairwise comparisons (Table 4) showed a significant difference between the metatarsal bar and plantar cover conditions (*p* = 0.003).

## Discussion

The aim of this study was to compare the effects of different forefoot pads on plantar pressures under the forefoot in older people with forefoot pain. Our results show that the metatarsal bar, plantar cover and metatarsal dome all significantly reduced forefoot peak pressure when compared to the control condition. This indicates that each of these forefoot pads reduce plantar pressure under the forefoot and may therefore be useful for the treatment of forefoot pain in older people.

In relation to forefoot peak pressure reduction, the plantar cover and the metatarsal dome positioned 5 mm distal to the metatarsal heads were the most effective forefoot pads. When the forefoot pads were compared, it was found that the metatarsal dome positioned 5 mm distal to the metatarsal heads was as effective as the plantar cover, while the metatarsal dome positioned 10 mm proximal to the metatarsal heads was as effective as the metatarsal bar (although both were less effective compared to the other two pads). Of note is that these two groups of forefoot pads demonstrated similar effects on peak pressure reductions despite their differences in design. Importantly, the only similarity between the forefoot pads to each other was the position of the pads relative to the plantar aspect of the metatarsal heads: the metatarsal dome positioned 5 mm distal and the plantar cover were both positioned distal to the metatarsal heads, while the metatarsal dome positioned 10 mm proximal and the metatarsal bar were both positioned proximal to the metatarsal heads. This most likely indicates that the position of pad relative to the plantar aspect of the metatarsal heads could be a more important factor in influencing forefoot peak pressure than the specific shape of the pad.

The mechanism of action of plantar forefoot pads is commonly thought to be through the redistribution of force over a larger area of the plantar surface of the foot [13,18-20]. From the results of this study, the mechanism of action in which the metatarsal dome positioned 10 mm proximal to the metatarsal heads, the metatarsal bar and the plantar cover reduced peak pressure can be attributed to an increase in forefoot contact area (since pressure = force/area). Due to the large forefoot mask used in this study, we were able to detect changes in forefoot contact area, and this is the first study to show significant differences in forefoot contact area with the use of different forefoot pads. Interestingly, a significant increase in contact area was not observed with the metatarsal dome positioned 5 mm distal to the metatarsal heads, even though it, and the plantar cover, provided the largest decrease in plantar pressure at the forefoot. To explain this finding further, the force readings need to be taken into account. All pads significantly reduced maximum force at the forefoot. Accordingly, in this study a combination of decreasing maximum force and increasing contact area is the mechanism by which the pads decreased plantar pressure at the forefoot. However, when interpreting the relationships between maximum force, contact area and peak pressure with the pedar®-X system, it is important to consider that these three variables may occur at different time points during the gait cycle.

Previous studies have investigated the effects of different forefoot pads [13,17] and different positions of metatarsal pads [21-23] on plantar pressure, but none of the studies have been conducted on older people in particular. One of the strengths of this study is that we specifically recruited older people that had either forefoot pain in the past or had forefoot pain at the time of the study. Accordingly, our findings can be generalised to community-dwelling older people with plantar forefoot pain. In addition, previous studies have not simultaneously investigated both the design of the forefoot pads and the different positions of the forefoot pads as we did in this study.

Further comparisons are difficult due to several design differences in our study compared with previous studies. Firstly, the focus of this study was on overall forefoot plantar pressure reduction and masking of individual metatarsal heads was avoided. We elected to take this approach because of poor reliability associated with masking of individual metatarsal heads [32] and the high prevalence of forefoot deformities in this population [1,5,6], possibly resulting in different degrees of forefoot anatomical variations between the participants. In addition, we wanted to be able to detect changes in contact area in the forefoot region due to the pads. Secondly, our study was a pragmatic study with a forefoot pad placement protocol that was designed to reflect clinical practice. Therefore, it is difficult to compare the results of the metatarsal dome positions with studies that utilised a protocol that involved pressure measurements for pad placement [22]. Thirdly, differences in forefoot pad design, terminology and placement of the pads have to be taken into account when comparing our results with previous studies [13,17].

One question that we were also interested in was whether the position of a metatarsal dome influences changes in plantar pressure reduction. Our findings demonstrated that positioning the metatarsal dome 5 mm distal to the metatarsal heads was more effective for reducing peak pressures over the forefoot compared to positioning the pad 10 mm proximal to the metatarsal heads. Although the plantar pressure reductions we measured for the plantar cover and the metatarsal dome positioned 5 mm distal to the metatarsal heads were relatively large, it is not known whether a 17 to 19% reduction in forefoot peak pressure is sufficient to reduce forefoot pain in older people. We did not assess forefoot pain levels when wearing the pads, as such data would be contaminated by the repeated measures design of the study and would not provide a valid indicator of pain relief provided by the pads over the longer term. Nevertheless, Kang et al. [14] reported pain relief associated with an 11.8% decrease in peak pressure when wearing a metatarsal pad, although they studied younger participants.

The metatarsal dome may be more feasible to use than the plantar cover in the clinical setting as it is readily available as a prefabricated pad and it takes up less space in the shoe. Since a large proportion of older people wear shoes that are too narrow for their feet [33], the metatarsal dome may allow for a better fit in shoes compared to the plantar cover. Therefore, we cautiously recommend the use of a metatarsal dome positioned 5 mm distal to the metatarsal heads in older people with forefoot pain. However, a randomised trial using patient-reported outcome measures needs to be undertaken before this recommendation can be supported with evidence. In particular, we did not measure long-term comfort of the pads, so this needs to be investigated in addition to variables such as pain reduction. It may be, for example, that a metatarsal dome positioned 5 mm distal to the metatarsal heads is not as comfortable as more proximally located pads and as a consequence, the more distal pad position may lead to adherence issues.

The findings from this study have to be viewed in light of four key limitations. First, the metatarsal bar and the plantar cover forefoot pads were fabricated by one of the investigators (PYL). Although the fabrication of these pads would not have been as consistent as the prefabricated metatarsal domes, several measures were put in place to ensure the consistency of the pads (refer to Additional file 1). Second, an inherent limitation of commercially-available in-shoe plantar pressure measurement systems is that they only measure the force perpendicular to the sensor surface [19,34]. Therefore, the effect of the forefoot pads on shear force cannot be evaluated with the system we used. Third, it cannot be assumed that the deformation of forefoot pads is uniform. Rather, it is likely that some degree of distortion occurs under load, resulting in some areas of the pad undergoing greater compression and adjacent areas less compression. The overall effect of this variability is uncertain and cannot be readily quantified with existing pressure systems. Finally, the limited spatial resolution of plantar pressure systems introduces an inherent degree of error in relation to contact area, and therefore, pressure measurements [35]. For platform systems incorporating sensors of 5 × 6 mm, the degree of error for the measurement of total foot contact area has been estimated at 11% [36], however we are unaware of any similar analyses of in-shoe systems. We did, however, use a repeated-measures research design, and Ramanathan et al. [30] have previously found that the pedar^®^-X demonstrated high repeatability under the metatarsal heads. Therefore, although the accuracy of *absolute* measurements of contact area could be questioned, *relative* comparisons between the padding trials within each participant are likely to be reasonably robust.

## Conclusions

Each of the forefoot pads evaluated in this study was effective for reducing forefoot peak pressure in older people with forefoot pain. However, the metatarsal dome positioned 5 mm distal to the metatarsal heads and the plantar cover were the most effective. Due to the availability of prefabricated metatarsal domes and their smaller size, we cautiously recommended the use of such pads, positioned 5 mm distal to the metatarsal heads, to reduce forefoot plantar pressures in older people with forefoot pain. Further comfort and pain assessment, alongside plantar pressure evaluation, in long-term clinical trials is now warranted.

## Competing interests

The authors have none to declare. HBM and KBL are Editor-in-Chief and Deputy Editor-in-Chief, respectively, of the *Journal of Foot and Ankle Research*. It is journal policy that editors are removed from the peer review and editorial decision making processes for papers they have co-authored.

## Authors’ contributions

KBL had full access to the data and takes responsibility for the integrity of the data and the accuracy of its analysis. Study concept and design: KBL, HBM and PYL. Acquisition of the data: PYL. Analysis and interpretation of the data: KBL, PYL, DRB and HBM. Drafting of manuscript: KBL, PYL, DRB and HBM. All authors read and approved the final manuscript.

## Supplementary Material

Additional file 1 Further details of forefoot pad fabrication and fitting. Click here for file
