# Supplementary material for: Comparison of the pressure-relieving properties of various types of forefoot pads in older people with forefoot pain
Source: J Foot Ankle Res. 2014 Mar 5;7:18. doi: 10.1186/1757-1146-7-18 (PMC4016518; doi:10.1186/1757-1146-7-18)
Supplement: Additional file 1 — Further details of forefoot pad fabrication and fitting. [file 1757-1146-7-18-S1.docx]

## Additional file 1

## Further details of forefoot pad fabrication and fitting

All of the *metatarsal bar* and *plantar cover* pads were fabricated for a range of shoe sizes (size 4 – 11) prior to the data collection session. To ensure consistency in size, a flat 4 mm polypropylene template was fabricated for each shoe size (Figure 1). The sizes of the pads were then based on that single template for that particular shoe size and the pads were fabricated accordingly using the template as a guide.

**Figure 1** The forefoot pads and the 4 mm polypropylene template used in their fabrication for shoe size 6. Left to right: (i) metatarsal bar, (ii) 4 mm polypropylene template, (iii) plantar cover.


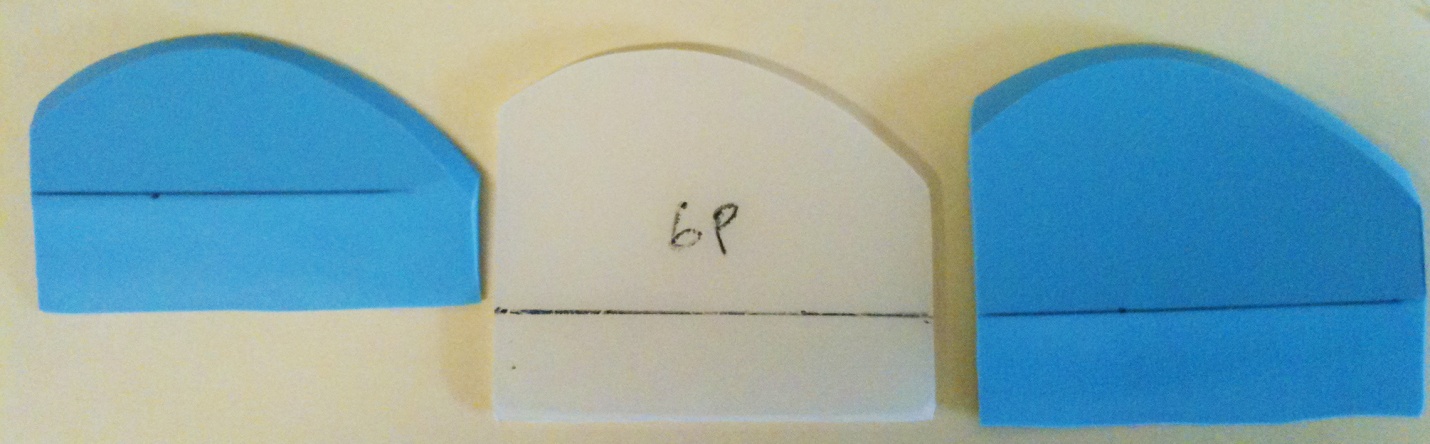


**(i)**

**(iii)**

**(ii)**

Note: all bevels were smoothed (i.e. the superior bevelled edges were rounded) prior to testing.

The plantar cover and metatarsal bar pads were fabricated with an approximately 30° proximal bevel of 25 mm and a 45° distal bevel of 10 mm (Figure 2).

**Figure 2** Side view of the fabricated forefoot pads showing the proximal and distal bevels. Left to right: (i) metatarsal bar, (ii) plantar cover.


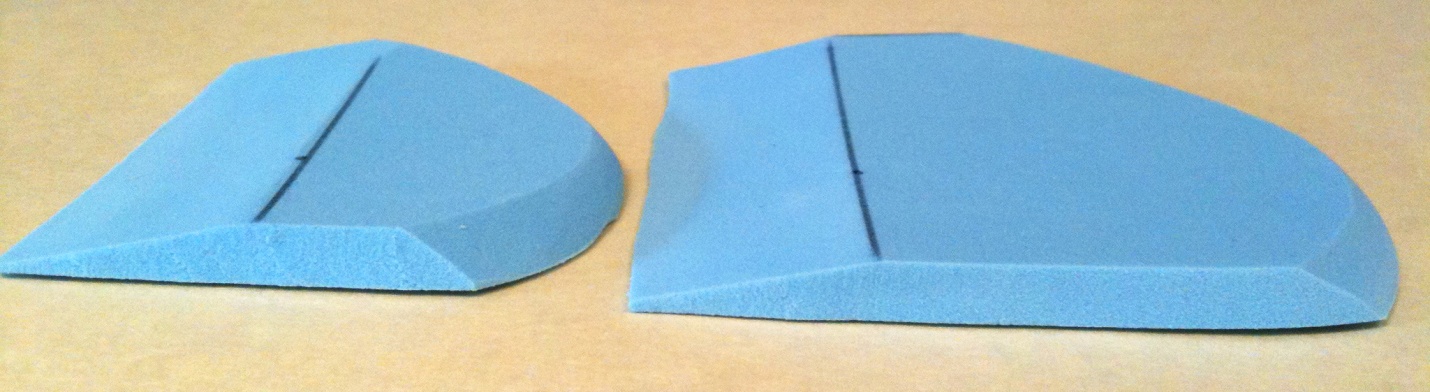


**(ii)**

**(i)**

Note: all bevels were smoothed (i.e. the superior bevelled edges were rounded) prior to testing.

Dimensions of the metatarsal bar pad were the same as the corresponding plantar cover pad of the same shoe size, with the exception that it was approximately 25 mm shorter in length. This was to enable positioning of the metatarsal bar just proximal to the metatarsal heads, rather than having full thickness of the pad under the metatarsal heads as is the case with a plantar cover. The shape of the metatarsal parabola was left and right foot specific and was the same for all the pads. The dimensions of the metatarsal bar pad was the same as the corresponding plantar cover pad, with the exception that it was approximately 25 mm shorter.

The forefoot pads were adhered using double-sided adhesive tape to the cardboard template to prevent the forefoot pad from moving during testing. To position the pads, the metatarsal heads of the participants were palpated and the centre of the most distal aspect of each metatarsal head was marked on the plantar surface of the foot using a pen. Following this, the template was positioned in the shoe and the participant’s foot was then placed in the shoe on top of the template without socks or stockings. After the shoe was fastened, the participants were instructed to stand to transfer the ink markings onto the template. The metatarsal parabola was then marked out on the template and the forefoot pads were positioned accordingly (Figure 1). To ensure consistency, determination of the metatarsal heads and the placement of the forefoot pads for all participants were undertaken by the same investigator (PYL).
